# Supplementary material for: Population-Specific Genetic and Non-Genetic Influences on Sleep Traits and Health Outcomes
Source: ArXiv. 2026 May 22:arXiv:2605.23521v1. Preprint. [Version 1] (PMC13229075)
Supplement: Supplement 1 [file NIHPP2605.23521v1-supplement-1.pdf]

Supplementary Materials

AoU recruitment and enrollment. The All of Us (AoU) Research program provides a large, comprehensive, and diverse biomedical dataset of participants in the United States. The goal of this program is to accelerate medical research and breakthroughs by enrolling at least one million individuals[10]. AoU began in May 2018 and, as of August 2024, more than 826,000 participants have been enrolled with over 80% from historically underrepresented groups in biomedical research. Participants provide AoU with a diverse set of data from Electronic Health Records (EHR), surveys, physical measurements, biospecimens, and wearable devices[15]. These data are organized into Curated Data Repositories (CDRs), which are divided into different access tiers based on the level of data detail available to researchers in the AoU Researcher Workbench. For our analyses, we used the Controlled Tier Dataset v7 CDR, which includes data from 413,457 participants enrolled from May 2018 to July 2022[10,14,15]. Data from participants enrolled after July 2022 were not available as of August 2024.

AoU datasets. We utilized five datasets from AoU Research Program – Genomic data, one-time Physical Measurement data, longitudinal EHR data, Fitbit sleep data, and All by All tables. *Genomic data:* The AoU's Controlled Tier provides access to participant genomic data in various formats. Our analyses focused on the short-read whole genome sequencing (srWGS) SNP and Indel callset, stored in the Hail VariantDataset (VDS) format. Using the provided table of samples flagged for relatedness, we removed 15,375 related participants from the 245,394 total participants with genomic data. Of the remaining 230,019 participants, demographic data were available for 230,013 participants. We excluded data from 4,713 participants (2.0%) with

sex at birth other than Male or Female and 8,806 participants (3.8%) with ethnicity other than Hispanic or non-Hispanic, leaving 212,529 participants (Table 1, eFigure 1).

Physical Measurement and EHR data: The Physical Measurement dataset contains one-time measurements taken at enrollment, while longitudinal data are available through the EHR dataset. Both datasets are standardized to the Observational Medical Outcomes Partnership (OMOP) common data model (CDM), in which medical information such as diagnoses, drugs, and procedures are mapped to standard concepts. We used AoU's Cohort Builder tool in the Researcher Workbench to filter participants with available WGS and EHR data. With this cohort, we pulled longitudinal data for hemoglobin A1C (HbA1C), fasting glucose, and insulin lab results, as well as height, weight, and waist circumference (WC) measurements from the EHR data using relevant concept IDs (eFigure 1). Additionally, one-time height, weight, and WC measurements taken at enrollment were extracted from the Physical Measurement (PM) dataset and pooled with the EHR data. BMI values were calculated from the height and weight measurements for each participant.

Fitbit sleep data: We used Fitbit-derived sleep duration data to calculate average sleep duration for participants with available Fitbit data in AoU. Of the 212,529 participants in our cohort, 7,655 had available sleep duration data. Daily sleep duration was calculated by summing up the minutes asleep for any sleep period classified as the 'main sleep' of the day. These values were then averaged to derive the average daily sleep duration for each participant.

Exclusion criteria for measurements: All lab and anthropometric measurements were filtered to exclude biologically implausible values. The following ranges were considered biologically implausible: >50% for HbA1C, >1000 mg/dL for fasting glucose, >100 µIU/mL for insulin, <111.8 cm or >228.6 cm for height[11,12], <24.9 kg or >453.6 kg for weight[11,12], and <5 kg/m^2 or

≥100 kg/m^2 for BMI[11,13]. To improve reliability of the BMI data, height and weight measurements underwent quality control following established methods in literature. In addition to filtering out implausible measurements, these methods included removing any height and weight measurements taken during inpatient and emergency department visits and any measurements taken within one year of a pregnancy-related diagnosis.[11,13] We then removed within-subject outliers, which were measurements for each participant that met the following criteria outlined by Cheng et al.[12]: weight measurements where (1) the range was >22.7 kg and the absolute difference between that specific weight and average weight was >70% of the range or (2) the standard deviation (SD) was >20% of the average weight and the absolute difference between that particular weight and average weight was greater than the SD; and height measurements where (1) the absolute difference between that particular height and average height was greater than the SD and (2) the SD was >2.5% of the average height. Any participants that still had a >5cm height change were removed. The remaining height and weight values were matched by measurement date and used to calculate BMI values for each participant. Finally, for all lab and anthropometric measurements, the median was used if a participant had multiple measurements on the same day. The full filtering process including participant counts can be seen in eFigure 1.

All by All Tables: As of June 2024, All by All tables were released to Controlled Tier users in the AoU Researcher Workbench. Leveraging srWGS and phenotypic data from ~250,000 participants in the AoU Researcher Workbench, these tables contain GWAS results for 8,895 high-quality ancestry group and phenotype pairs. The ancestry groups included were African (AFR), Admixed American (AMR), European (EUR), East Asian (EAS), South Asian (SAS), and Middle Eastern (MID). In total, there were 3,414 unique phenotypes of six categories:

physical measurements, lab measurements, phecodes, phecodeX, personal and family health history, and EHR sourced drugs and medications. In addition to results by ancestry, results from a meta-analysis that combined ancestry-specific results were also available.

PRS calculation. Weights used in the PRS calculation were the reported effect sizes from published GWAS, which were odds ratios (OR) for morning chronotype[8], OR for short sleep[9], and beta values representing 1-minute increments for sleep duration.[9] OR values were transformed to log OR prior to calculation. Because the reported SNP locations were relative to the GRCh37 reference genome and AoU dataset uses GRCh38, we employed the pyliftover Python package to convert the GRCh37 positions to GRCh38. Utilizing the AoUPRS package[19], GWAS SNPs were matched to those in AoU by genomic location and PRS was calculated for each sleep trait by summing the number of effect alleles in an individual weighted by their effect size.

Formulas used in cross-sectional and longitudinal analyses

Cross-sectional analysis: We performed multivariable linear regression for each pair of sleep trait PRS and measurement as follows, where β represents the coefficients and ε denotes the error term:

$$\begin{aligned}\text{Measurement} = {} & \beta_0 + \beta_1(\text{PRS}) + \beta_2(\text{smoking}) + \beta_3(\text{obesity}) + \beta_4(\text{diabetes}) + \beta_5(\text{age}) \\ & + \beta_6(\text{ancestry}) + \beta_7(\text{ethnicity}) + \beta_8(\text{sex}) + \varepsilon\end{aligned}$$

All continuous variables were standardized to a mean of 0 and a standard deviation of 1. We assessed multicollinearity among independent variables using the variance inflation factor (VIF) and iteratively excluded covariates with high VIF until all variables had VIF less than 10.

Longitudinal analysis: We employed linear mixed models to account for the correlation between longitudinal measurements within individuals while assessing the association between each sleep trait PRS and measurement pair. In this linear mixed models, we included random intercepts to account for individual-level variation in baseline outcomes and random slopes for age at measurement, allowing the relationship between age at measurement and each outcome to vary across participants. PRS was modeled as a fixed effect and, along with covariates, was estimated via conditional likelihood. The formulation is as follows, where b_{0i} is the random intercept and b_{1i} is the random slope for each participant *i*.

$$\begin{aligned}\text{Measurement}_{\text{ij}} &= \beta_0 + \beta_1(\text{age at measurement}_{ij}) + \beta_2(\text{PRS}_i) + \beta_3(\text{smoking}_i) \\ &+ \beta_4(\text{obesity}_i) + \beta_5(\text{diabetes}_i) + \beta_6(\text{ancestry}_i) + \beta_7(\text{ethnicity}_i) + \beta_8(\text{sex}_i) \\ &+ b_{0i} + b_{1i}(\text{age at measurement}_{ij}) + \varepsilon_{ij}\end{aligned}$$

We included the same covariates in the longitudinal analyses as in the cross-sectional analyses. Standardization of continuous variables and handling of multicollinearity aligned with the cross-sectional analyses as well.

Genetic associations with sleep traits and their implications for disease pathways

We examined the genes associated with specific SNPs linked to various health phenotypes in our study and investigated their roles in established disease pathways. For example, the chronotype SNP rs1421085 (chr16:53767042) is located within the FTO gene region, a well-known predictor of polygenic obesity—a significant risk factor for type 2 diabetes and other metabolic disorders. Similarly, the sleep duration SNP rs9940646 (chr16:53766717), which in our analysis was associated with phenotypes related to obesity, diabetes, and cardiovascular conditions, is

also situated within the FTO gene region, further highlighting FTO's central role in obesity-related traits. Another notable example is the short sleep SNP rs2820313 (chr1:201901093), a mutation in the leiomodin1 (LMOD1) gene, a smooth muscle-restricted and relatively understudied gene.[19] In our study, this SNP demonstrated significant associations with the use of antiepileptic medications, BMI, and type 2 diabetes (Figure 2). These findings align with the known relationship between sleep patterns and seizure activity, as sleep deprivation can trigger seizures in individuals with epilepsy[20] – a condition often associated with obesity and diabetes.[21] Additionally, recent studies have implicated LMOD1 in nodding syndrome, a form of pediatric epilepsy, suggesting that antibodies against LMOD1 may contribute to its pathogenesis.[22] The chronotype SNP rs113851554 (chr2:66523432), located in the MES1 gene, showed a significant association with sleep disorders and dopamine-related medications (e.g., dopamine agonists, dopaminergic agents), but only within the EUR ancestry group. Previous studies have reported that the MES1 GWAS signals were among the strongest genetic associations reported for the development of restless legs syndrome, a common sleep-related disorder.[23,24]

eFigure 1. Participant inclusion criteria flow diagram.
eFigure 2. GWAS by ancestry groups.
eFigure 3. Correlation between PRS-SS and actual sleep duration measurements from Fitbit data (left) and between PRS-SD and actual sleep duration measurements from Fitbit data (right).
eTable 1. List of concept IDs, ICD codes, and names used to query data.
eTable 2. Grouping of phenotypes associated with sleep trait SNPs identified in meta-analysis and ancestry-specific analyses
eTable 3. Cross-sectional analyses results by ancestry
eTable 4. Longitudinal analyses results by ancestry
eTable 5. Cross-sectional analyses results by ancestry, adjusted for Fitbit-measured sleep duration
eTable 6. Longitudinal analyses results by ancestry, adjusted for Fitbit-measured sleep duration

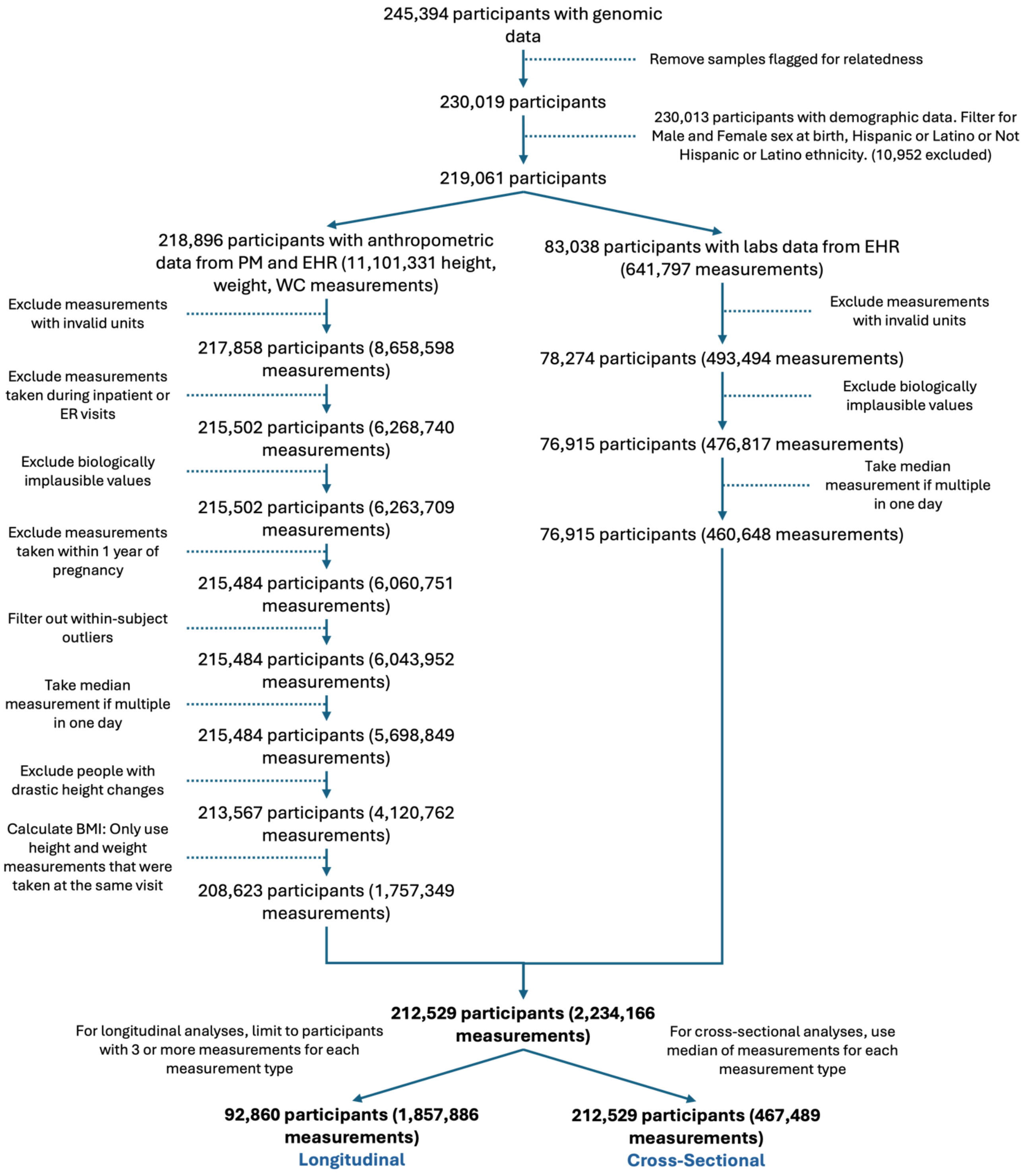

eFigure 1. Participant inclusion criteria flow diagram. A flow diagram showing the filtering criteria and the number of participants and measurements remaining after each step.

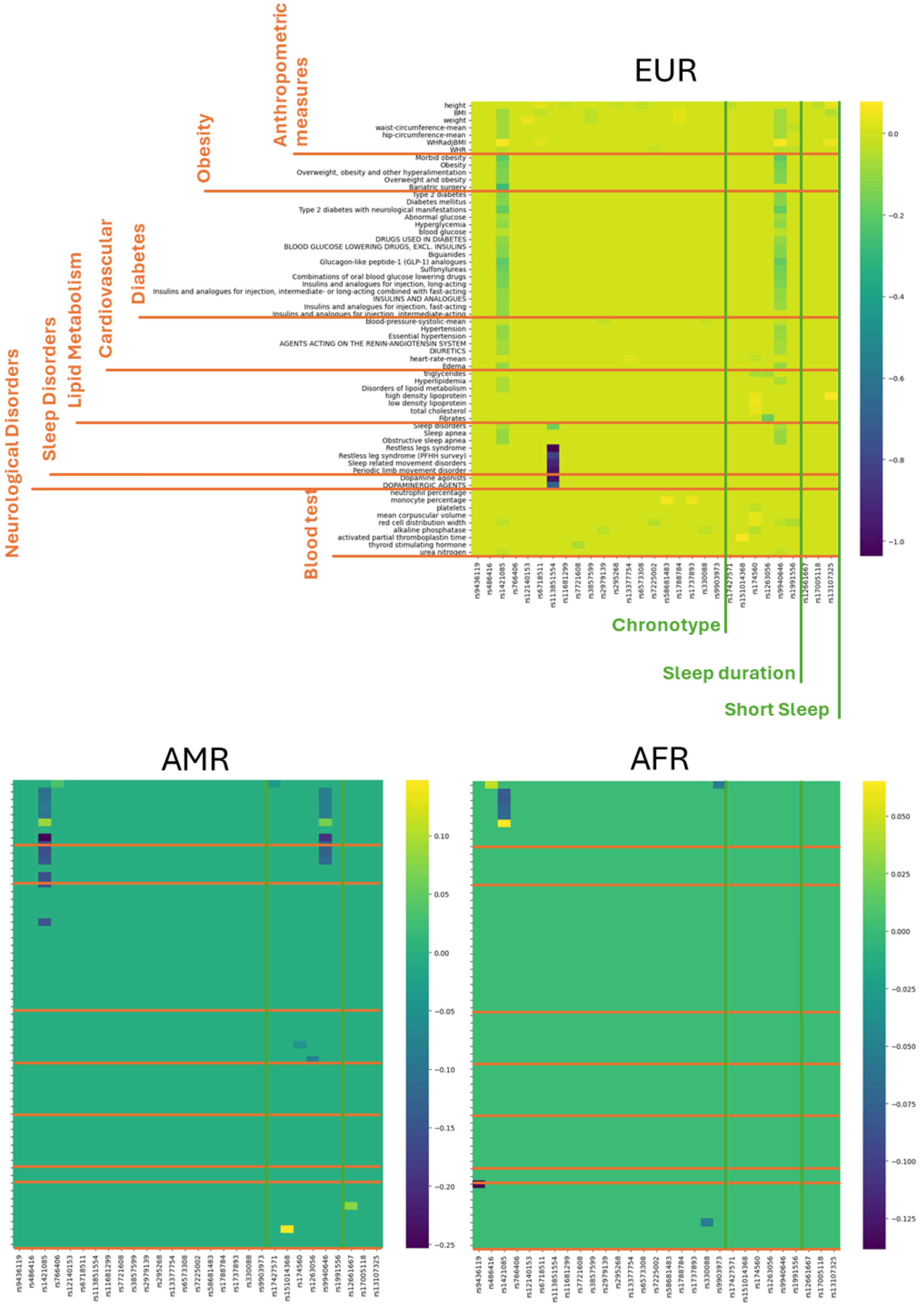

eFigure 2. GWAS by ancestry groups. A heatmap presenting the effect sizes for associations between sleep trait SNPs and phenotypes.

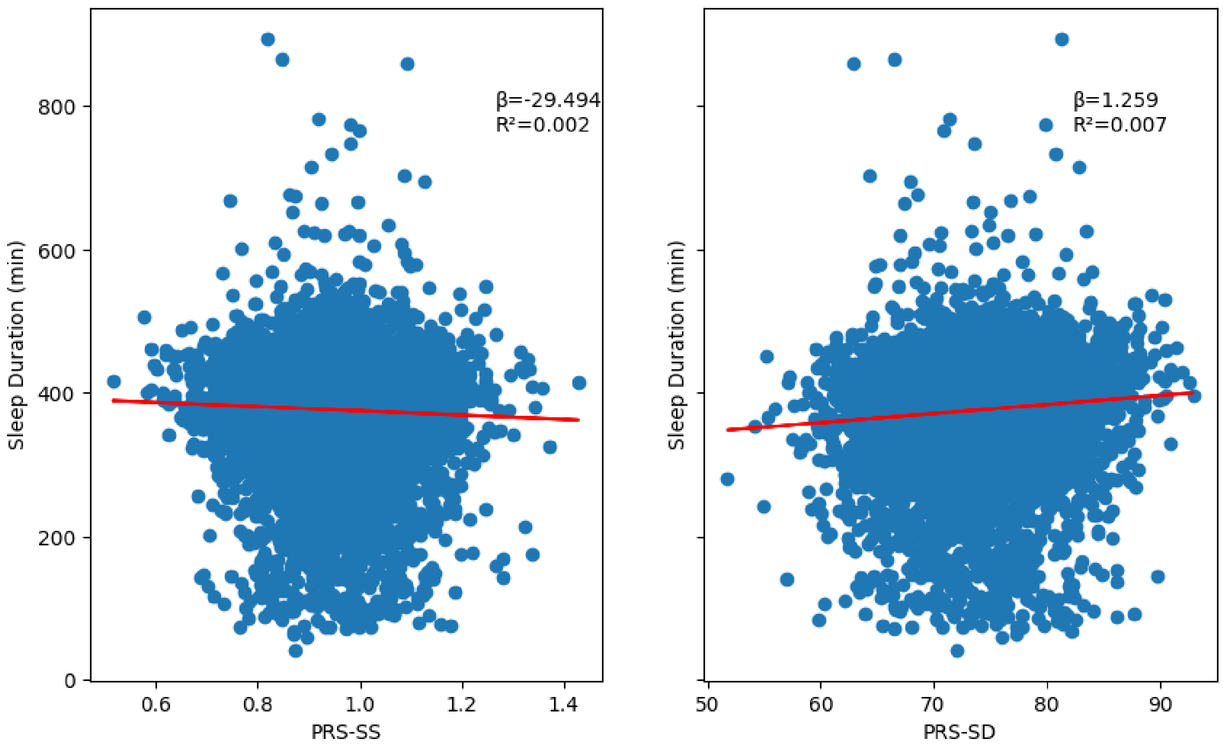

eFigure 3. Correlation between PRS-SS and actual sleep duration measurements from Fitbit data (left) and between PRS-SD and actual sleep duration measurements from Fitbit data (right).

eTable 1. List of concept IDs, concept names, and ICD codes used to query data.

Measurement/Condition	Query Value
Height	Concept ID: 903133, 3019171, 3023540, 3036277
Weight	Concept ID: 903121, 3013762, 3023166, 3025315, 3027492
Waist Circumference	Concept ID: 903124
Hemoglobin A1C	Concept ID: 300309, 3004410, 3005673, 3007263, 4184637, 4197971, 40762352, 42869630
Fasting glucose	Concept ID: 3036671, 3037110, 3037187, 4182052, 46235168
Insulin	Concept ID: 3016244, 3022466, 36303996
Obesity	Concept ID: 433736 and all descendant concepts
Diabetes	Concept ID: 201820 and all descendant concepts<br><br>Concept names for antidiabetic drugs:<br>• Type 1 diabetes: insulin, pramlintide, insulin glulisine, insulin lispro, insulin aspart, insulin glargine, insulin detemir, insulin degludec, insulin NPH<br>• Type 2 diabetes: acetohexamide, tolazamide, chlorpropamide, glipizide, glyburide, glimepiride, repaglinide, nateglinide, metformin, rosiglitazone, pioglitazone, troglitazone, acarbose, miglitol, sitagliptin, exenatide, saxagliptin, linagliptin, liraglutide, semaglutide, canagliflozin, dapagliflozin, empagliflozin, alogliptin, colesevelam, albiglutide, dulaglutide, lixisenatide
Smoking	Concept ID: 440612, 4146763, 4103418, 4209423, 37110444, 37109023, 437264, 36716478, 765451, 3654548, 36716473 and all descendant concepts
Pregnancy	ICD-9 Code: 630-679.14<br><br>ICD-10 Code: O0.0-O9A53, Z33.X, Z34.X, Z3A.X, Z37.X, Z38.X, Z39.0, A34.X

eTable 2. Grouping of phenotypes associated with sleep trait SNPs identified in meta-analysis and ancestry-specific analyses

Phenotype group	Phenotypes	
	**Meta-analysis**	**Ancestry-specific analysis**
Anthropometric Measures	'waist-circumference-mean', 'WHRadjBMI', 'weight','BMI', 'hip-circumference-mean', 'WHR'	'height', 'BMI', 'weight', 'waist-circumference-mean', 'hip-circumference-mean', 'WHRadjBMI', 'WHR'
Obesity and Related Conditions	'Obesity', 'Overweight and obesity'	'Morbid obesity', 'Obesity', 'Overweight, obesity and other hyperalimentation', 'Overweight and obesity', 'Bariatric surgery',
Diabetes and Blood Glucose Management	'Type 2 diabetes', 'Type 1 diabetes', 'Insulins and analogues for injection, long-acting', 'Insulins and analogues for injection, intermediate-acting', 'Diabetic retinopathy', 'Abnormal glucose', 'Diabetes mellitus'	'Type 2 diabetes', 'Diabetes mellitus', 'Type 2 diabetes with neurological manifestations', 'Abnormal glucose', 'Hyperglycemia', 'blood glucose', 'DRUGS USED IN DIABETES', 'BLOOD GLUCOSE LOWERING DRUGS, EXCL. INSULINS', 'Biguanides', 'Glucagon-like peptide-1 (GLP-1) analogues', 'Sulfonylureas', 'Combinations of oral blood glucose lowering drugs', 'Insulins and analogues for injection, long-acting', 'Insulins and analogues for injection, intermediate- or long-acting combined with fast-acting', 'INSULINS AND ANALOGUES', 'Insulins and analogues for injection, fast-acting', 'Insulins and analogues for injection, intermediate-acting'
Cardiovascular and Hypertension-Related	'blood-pressure-diastolic-mean', 'AGENTS ACTING ON THE RENIN-ANGIOTENSIN SYSTEM'	'blood-pressure-systolic-mean', 'Hypertension', 'Essential hypertension', 'AGENTS ACTING ON THE RENIN-ANGIOTENSIN SYSTEM', 'DIURETICS', 'heart-rate-mean', 'Edema'
Lipid Metabolism	'Hyperlipidemia'	'triglycerides', 'Hyperlipidemia', 'Disorders of lipoid metabolism', 'high density lipoprotein', 'low density lipoprotein', 'total cholesterol', 'Fibrates'
Sleep Disorders	NA	'Sleep disorders', 'Sleep apnea', 'Obstructive sleep apnea', 'Restless legs syndrome', 'Restless leg syndrome (PFHH survey)', 'Sleep related movement disorders', 'Periodic limb movement disorder'

Phenotype group	Phenotypes	
	**Meta-analysis**	**Ancestry-specific analysis**
Neurological Disorders and Medications	'ANTIEPILEPTICS'	'Dopamine agonists', 'DOPAMINERGIC AGENTS'
Hematological and Laboratory Values	'red cell distribution width', 'monocyte percentage', 'platelets'	'neutrophil percentage', 'monocyte percentage', 'platelets', 'mean corpuscular volume', 'red cell distribution width', 'alkaline phosphatase', 'activated partial thromboplastin time', 'thyroid stimulating hormone', 'urea nitrogen'

eTable 3. Cross-sectional analyses results by ancestry

Genetic Ancestry	N	%	Measurement	N	%	PRS-C β	PRS-C 95% CI	PRS-C p-value	PRS-SS β	PRS-SS 95% CI	PRS-SS p-value	PRS-SD β	PRS-SD 95% CI	PRS-SD p-value
All	212,529	100.0%	BMI	190,876	89.8%	0.002	[-0.003, 0.007]	0.424	0.012***	[0.008, 0.017]	<0.001	-0.022***	[-0.027, -0.017]	<0.001
			WC	192,979	90.8%	0.005*	[0.001, 0.01]	0.014	0.011***	[0.007, 0.015]	<0.001	-0.016***	[-0.02, -0.011]	<0.001
			Fasting glucose	7,372	3.5%	-0.003	[-0.026, 0.02]	0.789	-0.007	[-0.028, 0.014]	0.529	0.001	[-0.022, 0.024]	0.919
			HbA1C	75,055	35.3%	-0.001	[-0.008, 0.006]	0.794	0.004	[-0.002, 0.011]	0.162	-0.007	[-0.014, 0.0]	0.055
			Insulin	1,207	0.6%	0.005	[-0.054, 0.064]	0.865	0.050	[-0.004, 0.104]	0.070	-0.070*	[-0.133, -0.006]	0.031
EUR	119,539	56.2%	BMI	106,895	50.3%	0.003	[-0.003, 0.009]	0.339	0.016***	[0.01, 0.022]	<0.001	-0.016***	[-0.022, -0.01]	<0.001
			WC	108,451	51.0%	0.007**	[0.002, 0.012]	0.004	0.013***	[0.008, 0.018]	<0.001	-0.014***	[-0.019, -0.009]	<0.001
			Fasting glucose	4,413	2.1%	-0.004	[-0.031, 0.023]	0.761	0.003	[-0.023, 0.03]	0.801	0.008	[-0.019, 0.034]	0.579
			HbA1C	43,747	20.6%	0.006	[-0.003, 0.014]	0.176	0.008	[-0.001, 0.016]	0.067	-0.007	[-0.015, 0.002]	0.114
			Insulin	624	0.3%	0.024	[-0.051, 0.099]	0.533	0.066	[-0.009, 0.14]	0.084	-0.066	[-0.14, 0.009]	0.085
AFR	47,212	22.2%	BMI	42,737	20.1%	-0.007	[-0.016, 0.002]	0.108	0.002	[-0.007, 0.011]	0.659	-0.020***	[-0.029, -0.011]	<0.001
			WC	43,828	20.6%	0.000	[-0.009, 0.008]	0.916	0.005	[-0.003, 0.014]	0.245	-0.009*	[-0.017, -0.0]	0.047
			Fasting glucose	1,397	0.7%	0.021	[-0.027, 0.069]	0.397	-0.023	[-0.071, 0.025]	0.347	-0.012	[-0.061, 0.036]	0.622
			HbA1C	16,140	7.6%	-0.005	[-0.019, 0.008]	0.451	-0.017*	[-0.031, -0.004]	0.013	0.006	[-0.008, 0.02]	0.387
			Insulin	371	0.2%	-0.008	[-0.109, 0.094]	0.881	0.036	[-0.065, 0.137]	0.487	-0.010	[-0.113, 0.093]	0.851
AMR	36,965	17.4%	BMI	33,037	15.5%	0.012*	[0.001, 0.023]	0.026	0.009	[-0.001, 0.02]	0.080	-0.032***	[-0.043, -0.021]	<0.001

			WC	32,721	15.4%	0.005	[-0.004, 0.015]	0.284	0.008	[-0.002, 0.018]	0.116	-0.021***	[-0.03, -0.011]	<0.001
			Fasting glucose	1,299	0.6%	-0.022	[-0.072, 0.028]	0.393	-0.026	[-0.076, 0.023]	0.298	0.000	[-0.05, 0.05]	0.993
			HbA1C	12,540	5.9%	-0.010	[-0.025, 0.005]	0.175	0.019*	[0.004, 0.033]	0.014	-0.013	[-0.028, 0.002]	0.094
			Insulin	168	0.1%	-0.099	[-0.247, 0.05]	0.191	0.029	[-0.12, 0.178]	0.701	-0.070	[-0.218, 0.077]	0.348
EAS	5,198	2.4%	BMI	4,905	2.3%	0.021	[-0.006, 0.048]	0.128	0.018	[-0.009, 0.044]	0.200	-0.012	[-0.039, 0.015]	0.375
			WC	4,681	2.2%	0.017	[-0.007, 0.041]	0.164	0.029*	[0.005, 0.053]	0.018	-0.017	[-0.041, 0.007]	0.163
			Fasting glucose	†	†	-0.113	[-0.247, 0.02]	0.096	0.160*	[0.029, 0.291]	0.017	-0.067	[-0.199, 0.064]	0.314
			HbA1C	1,428	0.7%	0.009	[-0.035, 0.053]	0.684	0.009	[-0.035, 0.053]	0.684	-0.015	[-0.059, 0.029]	0.500
			Insulin	†	†	0.256	[-0.308, 0.819]	0.332	0.160	[-0.442, 0.762]	0.562	-0.007	[-0.617, 0.603]	0.981
SAS	2,785	1.3%	BMI	2,571	1.2%	0.025	[-0.012, 0.062]	0.187	0.033	[-0.004, 0.071]	0.081	0.008	[-0.029, 0.046]	0.664
			WC	2,559	1.2%	0.004	[-0.028, 0.035]	0.816	0.021	[-0.01, 0.052]	0.183	0.009	[-0.022, 0.04]	0.572
			Fasting glucose	†	†	0.003	[-0.221, 0.227]	0.981	0.061	[-0.158, 0.279]	0.580	-0.172	[-0.392, 0.048]	0.124
			HbA1C	879	0.4%	0.004	[-0.051, 0.058]	0.894	0.048	[-0.007, 0.102]	0.088	-0.036	[-0.091, 0.019]	0.202
			Insulin	†	†	0.280	[-0.66, 1.22]	0.525	-0.001	[-0.783, 0.782]	0.998	-0.215	[-0.894, 0.464]	0.501
MID	830	0.4%	BMI	731	0.3%	-0.022	[-0.093, 0.049]	0.546	0.039	[-0.031, 0.11]	0.275	-0.002	[-0.073, 0.068]	0.946
			WC	739	0.3%	0.002	[-0.056, 0.059]	0.957	0.044	[-0.013, 0.101]	0.127	-0.006	[-0.062, 0.051]	0.849
			Fasting glucose	†	†	-0.265	[-0.668, 0.137]	0.183	-0.302	[-0.661, 0.057]	0.094	0.427*	[0.1, 0.754]	0.013
			HbA1C	321	0.2%	-0.060	[-0.152, 0.033]	0.209	0.052	[-0.04, 0.145]	0.267	-0.016	[-0.108, 0.076]	0.736
			Insulin	†	†	-0.365	[-1.525, 0.795]	0.309	0.459	[-1.407, 2.325]	0.401	-0.304	[-0.995, 0.386]	0.199

Coefficients (β) from ordinary least squares (OLS) multivariable linear regression analysis of PRS for chronotype, short sleep, sleep duration on BMI, WC, fasting glucose, insulin, and hemoglobin A1C (HbA1C), adjusted for age, ancestry, ethnicity, sex, smoking, obesity, and diabetes. PRS and all measurements were standardized to a mean of 0 and a standard deviation of 1. Percentages are of the total 212,529 participants. Significance levels: *p < 0.05, **p < 0.01, ***p < 0.001. † Counts suppressed per the All of Us Data and Statistics Dissemination Policy.

eTable 4. Longitudinal analyses results by ancestry

						PRS-C			PRS-SS			PRS-SD		
**Genetic Ancestry**	**N**	**%**	**Measurement**	**N**	**%**	**β**	**95% CI**	**p-value**	**β**	**95% CI**	**p-value**	**β**	**95% CI**	**p-value**
All	92,860	43.7%	BMI	74,094	34.9%	0.002	[-0.007, 0.011]	0.637	0.017***	[0.008, 0.025]	<0.001	-0.016***	[-0.026, -0.007]	<0.001
			WC	0	0.0%	N/A	N/A	N/A	N/A	N/A	N/A	N/A	N/A	N/A
			Fasting glucose	1,728	0.8%	0.009	[-0.023, 0.041]	0.573	-0.016	[-0.044, 0.012]	0.262	0.028	[-0.004, 0.061]	0.084
			HbA1C	39,233	18.5%	-0.003	[-0.011, 0.005]	0.469	0.003	[-0.005, 0.011]	0.430	-0.005	[-0.013, 0.004]	0.290
			Insulin	135	0.1%	0.091	[-0.042, 0.224]	0.181	0.134*	[0.008, 0.259]	0.037	-0.007	[-0.109, 0.095]	0.890
EUR	60,723	28.6%	BMI	50,573	23.8%	0.003	[-0.008, 0.013]	0.607	0.017**	[0.006, 0.027]	0.002	-0.014**	[-0.024, -0.003]	0.010
			WC	0	0.0%	N/A	N/A	N/A	N/A	N/A	N/A	N/A	N/A	N/A
			Fasting glucose	910	0.4%	0.020	[-0.023, 0.063]	0.368	0.013	[-0.03, 0.056]	0.549	0.029	[-0.014, 0.073]	0.190
			HbA1C	22,387	10.5%	0.003	[-0.007, 0.012]	0.576	0.008	[-0.002, 0.017]	0.122	-0.005	[-0.015, 0.004]	0.279
			Insulin	63	0.0%	0.081	[-0.096, 0.258]	0.372	0.229**	[0.072, 0.386]	0.004	-0.125*	[-0.247, -0.003]	0.045
AFR	16,505	7.8%	BMI	11,314	5.3%	-0.011	[-0.096, 0.258]	0.331	0.009	[-0.013, 0.031]	0.421	-0.010	[-0.032, 0.012]	0.379
			WC	0	0.0%	N/A	N/A	N/A	N/A	N/A	N/A	N/A	N/A	N/A
			Fasting glucose	467	0.2%	0.026	[-0.031, 0.082]	0.373	-0.043	[-0.098, 0.012]	0.128	0.043	[-0.013, 0.098]	0.132
			HbA1C	9,261	4.4%	-0.001	[-0.018, 0.015]	0.869	-0.018*	[-0.035, -0.002]	0.030	0.011	[-0.005, 0.027]	0.178

			Insulin	39	0.0%	0.059	[-0.085, 0.203]	0.419	0.104	[-0.076, 0.284]	0.259	-0.003	N/A	N/A
			BMI	9,212	4.3%	0.018	[-0.007, 0.043]	0.156	0.021	[-0.004, 0.046]	0.100	-0.030*	[-0.056, -0.004]	0.024
			WC	0	0.0%	N/A	N/A	N/A	N/A	N/A	N/A	N/A	N/A	N/A
			Fasting glucose	302	0.1%	-0.004	[-0.083, 0.075]	0.922	-0.055	[-0.136, 0.026]	0.183	0.031	[-0.049, 0.111]	0.453
			HbA1C	6,274	3.0%	-0.014	[-0.034, 0.006]	0.158	0.013	[-0.007, 0.033]	0.192	-0.019	[-0.04, 0.001]	0.060
AMR	12,176	5.7%	Insulin	22	0.0%	0.376	[-0.067, 0.818]	0.096	0.085	[-0.441, 0.61]	0.752	0.205	[-0.115, 0.526]	0.209
			BMI	1,745	0.8%	-0.007	[-0.06, 0.046]	0.793	-0.018	[-0.069, 0.033]	0.497	-0.033	[-0.083, 0.017]	0.196
			WC	0	0.0%	N/A	N/A	N/A	N/A	N/A	N/A	N/A	N/A	N/A
			Fasting glucose	†	†	-0.076	[-0.167, 0.016]	0.105	0.194	[-0.145, 0.534]	0.262	0.167**	[0.055, 0.278]	0.003
			HbA1C	700	0.3%	-0.040	[-0.094, 0.013]	0.139	0.012	[-0.041, 0.066]	0.651	0.006	[-0.047, 0.059]	0.833
EAS	1,970	0.9%	Insulin	†	†	0.087	[-8.596, 8.77]	0.984	-0.482	N/A	N/A	0.683	[-2.815, 4.182]	0.702
			BMI	952	0.4%	0.023	[-0.05, 0.095]	0.540	0.038	[-0.033, 0.11]	0.292	-0.015	[-0.086, 0.056]	0.680
			WC	0	0.0%	N/A	N/A	N/A	N/A	N/A	N/A	N/A	N/A	N/A
			Fasting glucose	†	†	0.069	[-0.161, 0.298]	0.558	-0.190	[-0.5, 0.12]	0.230	0.139	[-0.335, 0.612]	0.566
			HbA1C	440	0.2%	-0.030	[-0.11, 0.049]	0.456	0.063	[-0.018, 0.145]	0.128	0.007	[-0.074, 0.088]	0.867
SAS	1,117	0.5%	Insulin	†	†	-0.184	N/A	N/A	-0.337	[-1.505, 0.83]	0.571	0.193	[-15.682, 16.068]	0.981
			BMI	298	0.1%	-0.013	[-0.129, 0.103]	0.829	-0.004	[-0.12, 0.111]	0.941	0.043	[-0.075, 0.16]	0.478
			WC	0	0.0%	N/A	N/A	N/A	N/A	N/A	N/A	N/A	N/A	N/A
MID	369	0.2%	Fasting glucose	†	†	0.512	N/A	N/A	0.471	[-0.261, 1.202]	0.207	-0.018	[-23.442, 23.407]	0.999

			HbA1C	†	†	-0.115	[-0.242, 0.012]	0.076	0.081	[-0.031, 0.193]	0.157	0.013	[-0.108, 0.133]	0.837
			Insulin	0	0.0%	N/A	N/A	N/A	N/A	N/A	N/A	N/A	N/A	N/A

Fixed effects coefficients (β) from longitudinal analyses of PRS for sleep traits on BMI, WC, fasting glucose, hemoglobin A1C (HbA1C), insulin. PRS and all measurements were standardized to a mean of 0 and a standard deviation of 1. The linear mixed models were adjusted for ancestry, ethnicity, sex, smoking, obesity, and diabetes. Percentages are of the total 212,529 participants. Confidence intervals and p-values were unable to be computed for some parameters due to insufficient sample size or data variability. Significance levels: *p < 0.05, **p < 0.01, ***p < 0.001. † Counts suppressed per the All of Us Data and Statistics Dissemination Policy.

eTable 5. Cross-sectional analyses results by ancestry, adjusted for Fitbit-measured sleep duration

						PRS-C			Fitbit Sleep Duration (PRS-C)‡			PRS-SS			Fitbit Sleep Duration (PRS-SS)‡			PRS-SD			Fitbit Sleep Duration (PRS-SD)‡		
**Genetic Ancestry**	**N**	**%**	**Measurement**	**N**	**%**	**β**	**95% CI**	**p-value**	**β**	**95% CI**	**p-value**	**β**	**95% CI**	**p-value**	**β**	**95% CI**	**p-value**	**β**	**95% CI**	**p-value**	**β**	**95% CI**	**p-value**
			BMI	7,091	3.3%	-0.006	[-0.029, 0.017]	0.599	-0.090***	[-0.112, -0.067]	0.000	0.012	[-0.01, 0.034]	0.281	-0.089***	[-0.112, -0.067]	0.000	-0.018	[-0.041, 0.005]	0.127	-0.089***	[-0.111, -0.066]	0.000
			WC	7,169	3.4%	0.003	[-0.017, 0.023]	0.760	-0.057***	[-0.077, -0.037]	0.000	0.005	[-0.014, 0.025]	0.587	-0.057***	[-0.076, -0.037]	0.000	-0.001	[-0.021, 0.019]	0.936	-0.057***	[-0.077, -0.037]	0.000
			Fasting glucose	340	0.2%	-0.041	[-0.141, 0.058]	0.415	-0.061	[-0.156, 0.034]	0.204	-0.037	[-0.131, 0.057]	0.437	-0.066	[-0.161, 0.028]	0.170	0.031	[-0.066, 0.128]	0.531	-0.064	[-0.159, 0.03]	0.183
			HbA1C	2,558	1.2%	0.010	[-0.025, 0.046]	0.568	-0.028	[-0.063, 0.007]	0.118	-0.027	[-0.061, 0.007]	0.119	-0.030	[-0.065, 0.005]	0.098	0.018	[-0.018, 0.054]	0.316	-0.029	[-0.064, 0.006]	0.103
All	7,655	3.6%	Insulin	61	<0.1%	0.085	[-0.241, 0.41]	0.603	0.319*	[0.051, 0.587]	0.021	0.089	[-0.201, 0.379]	0.541	0.309*	[0.042, 0.577]	0.024	0.137	[-0.161, 0.436]	0.360	0.334*	[0.064, 0.603]	0.016

			BMI	5,994	2.8%	-0.006	[-0.03, 0.019]	0.653	-0.091***	[-0.116, -0.067]	0.000	0.018	[-0.007, 0.042]	0.157	-0.091***	[-0.115, -0.066]	0.000	-0.017	[-0.041, 0.007]	0.167	-0.090***	[-0.115, -0.066]	0.000
			WC	6,075	2.9%	0.000	[-0.022, 0.021]	0.967	-0.057***	[-0.078, -0.035]	0.000	0.010	[-0.011, 0.031]	0.343	-0.056***	[-0.078, -0.035]	0.000	-0.001	[-0.022, 0.021]	0.954	-0.057***	[-0.078, -0.035]	0.000
			Fasting glucose	287	0.1%	-0.044	[-0.146, 0.059]	0.401	-0.082	[-0.185, 0.021]	0.117	-0.040	[-0.141, 0.062]	0.443	-0.088	[-0.19, 0.015]	0.093	0.054	[-0.049, 0.156]	0.302	-0.085	[-0.187, 0.018]	0.104
			HbA1C	2,166	1.0%	0.016	[-0.021, 0.053]	0.386	-0.036	[-0.073, 0.002]	0.063	-0.023	[-0.06, 0.014]	0.216	-0.037*	[-0.075, -0.0]	0.050	0.009	[-0.028, 0.046]	0.644	-0.037	[-0.074, 0.001]	0.055
EUR	6,494	3.1%	Insulin	43	<0.1%	0.131	[-0.22, 0.482]	0.452	0.282	[-0.041, 0.606]	0.085	0.033	[-0.321, 0.388]	0.849	0.271	[-0.056, 0.599]	0.101	0.132	[-0.203, 0.468]	0.428	0.305	[-0.027, 0.636]	0.071
			BMI	397	0.2%	-0.006	[-0.102, 0.09]	0.901	-0.020	[-0.118, 0.078]	0.695	-0.036	[-0.134, 0.063]	0.473	-0.023	[-0.121, 0.076]	0.652	-0.018	[-0.115, 0.079]	0.717	-0.018	[-0.116, 0.081]	0.721
			WC	409	0.2%	0.041	[-0.045, 0.127]	0.348	-0.002	[-0.089, 0.086]	0.970	-0.062	[-0.149, 0.025]	0.161	-0.004	[-0.092, 0.083]	0.922	0.018	[-0.069, 0.104]	0.689	-0.001	[-0.088, 0.087]	0.990
			Fasting glucose	†	†	0.063	[-0.321, 0.447]	0.734	0.062	[-0.347, 0.471]	0.755	-0.023	[-0.541, 0.496]	0.927	0.065	[-0.35, 0.48]	0.744	-0.302	[-0.768, 0.164]	0.189	0.127	[-0.271, 0.525]	0.509
AFR	426	0.2%	HbA1C	180	0.1%	-0.042	[-0.175,	0.532	0.052	[-0.083,	0.445	-0.052	[-0.186,	0.446	0.044	[-0.091,	0.520	-0.009	[-0.144,	0.895	0.051	[-0.086,	0.465

							0.091]			0.187]			0.082]			0.179]			0.126]			0.187]	
			Insulin	†	†	-0.345	[-1.855, 1.164]	0.560	0.915	[-0.192, 2.023]	0.083	0.327	[-0.366, 1.021]	0.279	0.603	[-0.032, 1.238]	0.059	-0.044	[-0.986, 0.897]	0.903	0.794	[-0.223, 1.812]	0.096
			BMI	412	0.2%	0.004	[-0.089, 0.097]	0.934	-0.153**	[-0.246, -0.061]	0.001	-0.008	[-0.1, 0.085]	0.871	-0.154**	[-0.247, -0.061]	0.001	-0.020	[-0.114, 0.073]	0.669	-0.150**	[-0.244, -0.057]	0.002
			WC	409	0.2%	0.015	[-0.07, 0.099]	0.733	-0.111*	[-0.195, -0.026]	0.011	-0.003	[-0.087, 0.081]	0.937	-0.110*	[-0.195, -0.025]	0.011	-0.030	[-0.115, 0.054]	0.481	-0.106*	[-0.191, -0.02]	0.016
			Fasting glucose	†	†	-0.539	[-1.227, 0.149]	0.112	-0.083	[-0.671, 0.505]	0.760	-0.033	[-0.597, 0.531]	0.899	-0.096	[-0.777, 0.585]	0.760	-0.174	[-0.812, 0.463]	0.556	-0.047	[-0.724, 0.631]	0.881
			HbA1C	134	0.1%	0.013	[-0.146, 0.172]	0.876	-0.005	[-0.16, 0.149]	0.946	-0.051	[-0.206, 0.105]	0.521	-0.006	[-0.16, 0.148]	0.937	0.106	[-0.049, 0.261]	0.177	-0.025	[-0.18, 0.13]	0.749
AMR	435	0.2%	Insulin	†	†	0.808	N/A	N/A	0.616	N/A	N/A	0.273	N/A	N/A	1.154	N/A	N/A	0.793	[-3.181, 4.768]	0.239	0.434	[-3.54, 4.408]	0.398
			BMI	168	0.1%	-0.011	[-0.16, 0.138]	0.887	-0.151*	[-0.301, -0.001]	0.048	0.050	[-0.099, 0.198]	0.511	-0.149	[-0.299, 0.001]	0.052	-0.114	[-0.264, 0.036]	0.134	-0.139	[-0.289, 0.011]	0.070
EAS	176	0.1%	WC	161	0.1%	0.039	[-0.095, 0.172]	0.569	-0.217**	[-0.35, -0.085]	0.001	0.022	[-0.111, 0.155]	0.742	-0.217**	[-0.35, -0.085]	0.001	-0.097	[-0.231, 0.038]	0.158	-0.203*	[-0.337, -0.07]	0.003

			Fasting glucose	†	†	0.376	[-3.921, 4.674]	0.466	0.325	[-3.47, 4.119]	0.473	0.146	[-1.062, 1.355]	0.655	0.228	[-0.98, 1.436]	0.502	-0.586	[-5.512, 4.339]	0.372	0.311	[-2.752, 3.375]	0.419
			HbA1C	†	†	-0.045	[-0.305, 0.214]	0.726	-0.013	[-0.278, 0.253]	0.923	0.149	[-0.105, 0.402]	0.243	-0.023	[-0.278, 0.232]	0.857	0.148	[-0.14, 0.436]	0.305	-0.071	[-0.343, 0.202]	0.602
			Insulin	†	†	0.000	N/A	N/A	0.000	N/A	N/A	0.000	N/A	N/A	0.000	N/A	N/A	0.000	N/A	N/A	0.000	N/A	N/A
			BMI	106	<0.1 %	-0.079	[-0.267, 0.109]	0.406	-0.194	[-0.39, 0.001]	0.051	-0.101	[-0.291, 0.089]	0.293	-0.186	[-0.38, 0.008]	0.060	0.175	[-0.008, 0.358]	0.060	-0.183	[-0.374, 0.009]	0.062
			WC	102	<0.1 %	-0.081	[-0.254, 0.092]	0.355	-0.011	[-0.19, 0.168]	0.901	-0.124	[-0.299, 0.05]	0.161	-0.009	[-0.187, 0.169]	0.920	0.186*	[0.021, 0.351]	0.028	-0.010	[-0.185, 0.165]	0.910
			Fasting glucose	†	†	0.500	N/A	N/A	-0.500	N/A	N/A	0.500	N/A	N/A	-0.500	N/A	N/A	0.500	N/A	N/A	-0.500	N/A	N/A
			HbA1C	†	†	0.045	[-0.39, 0.48]	0.832	-0.132	[-0.597, 0.334]	0.563	-0.034	[-0.522, 0.453]	0.886	-0.123	[-0.589, 0.342]	0.588	0.317	[-0.101, 0.735]	0.130	-0.090	[-0.531, 0.351]	0.676
SAS	†	†	Insulin	†	†	0.000	N/A	N/A	0.000	N/A	N/A	0.000	N/A	N/A	0.000	N/A	N/A	0.000	N/A	N/A	0.000	N/A	N/A
			BMI	†	†	0.076	[-0.59, 0.741]	0.796	0.067	[-0.498, 0.631]	0.788	0.080	[-0.476, 0.636]	0.744	0.082	[-0.474, 0.637]	0.739	-0.169	[-0.699, 0.362]	0.477	0.059	[-0.482, 0.6]	0.803
			WC	†	†	0.234	[-0.567, 1.034]	0.487	-0.415	[-1.16, 0.33]	0.212	0.008	[-0.952, 0.967]	0.985	-0.404	[-1.23, 0.423]	0.265	0.164	[-0.572, 0.9]	0.592	-0.419	[-1.183, 0.344]	0.217
MID	†	†	Fasting glucose	†	†	0.000	N/A	N/A	0.000	N/A	N/A	0.000	N/A	N/A	0.000	N/A	N/A	0.000	N/A	N/A	0.000	N/A	N/A

			HbA1C	†	†	0.668	N/A	N/A	1.277	N/A	N/A	0.368	N/A	N/A	1.117	N/A	N/A	-0.312	N/A	N/A	1.064	N/A	N/A
			Insulin	0	0.0%	N/A	N/A	N/A	N/A	N/A	N/A	N/A	N/A	N/A	N/A	N/A	N/A	N/A	N/A	N/A	N/A	N/A	N/A

Coefficients (β) from ordinary least squares (OLS) multivariable linear regression analysis of PRS for chronotype, short sleep, sleep duration on BMI, WC, fasting glucose, insulin, and hemoglobin A1C (HbA1c), adjusted for Fitbit-derived sleep duration, age, ancestry, ethnicity, sex, smoking, obesity, and diabetes. PRS and all measurements were standardized to a mean of 0 and a standard deviation of 1. Percentages are of the total 212,529 participants. Significance levels: *p < 0.05, **p < 0.01, ***p < 0.001. † Counts suppressed per the All of Us Data and Statistics Dissemination Policy. ‡ Association test between Fitbit-derived sleep duration and health outcomes, with PRS included as a covariate..

eTable 6. Longitudinal analyses results by ancestry, adjusted for Fitbit-measured sleep duration

						PRS-C			Fitbit Sleep Duration (PRS-C)‡			PRS-SS			Fitbit Sleep Duration (PRS-SS)‡			PRS-SD			Fitbit Sleep Duration (PRS-SD)‡		
**Genetic Ancestry**	**N**	**%**	**Measurement**	**N**	**%**	**β**	**95% CI**	**p-value**	**β**	**95% CI**	**p-value**	**β**	**95% CI**	**p-value**	**β**	**95% CI**	**p-value**	**β**	**95% CI**	**p-value**	**β**	**95% CI**	**p-value**
All	4,078	1.9%	BMI	3,641	1.7%	0.006	[-0.036, 0.047]	0.790	-0.024	[-0.065, 0.018]	0.261	0.025	[-0.015, 0.065]	0.225	-0.023	[-0.065, 0.018]	0.268	-0.041	[-0.082, 0.001]	0.053	-0.022	[-0.063, 0.02]	0.308
			WC	0	0.0%	N/A	N/A	N/A	N/A	N/A	N/A	N/A	N/A	N/A	N/A	N/A	N/A	N/A	N/A	N/A	N/A	N/A	N/A
			Fasting glucose	†	†	-0.138	[-0.329, 0.052]	0.155	-0.055	[-0.213, 0.103]	0.493	0.008	[-0.136, 0.152]	0.913	-0.077	[-0.239, 0.086]	0.356	0.053	[-0.134, 0.24]	0.578	-0.067	[-0.235, 0.101]	0.435
			Hemoglobin A1C	1,318	0.6%	-0.006	[-0.044, 0.032]	0.754	-0.035	[-0.073, 0.002]	0.066	-0.009	[-0.045, 0.027]	0.625	-0.036	[-0.074, 0.002]	0.064	-0.004	[-0.042, 0.035]	0.851	-0.035	[-0.073, 0.003]	0.070
			Insulin	†	†	0.287	[-0.375, 0.948]	0.396	-0.042	[-0.836, 0.753]	0.918	0.140	N/A	N/A	-0.039	N/A	N/A	-0.082	[-0.411, 0.247]	0.626	0.074	[-0.365, 0.512]	0.742
EUR	3,560	1.7%	BMI	3,198	1.5%	0.007	[-0.037,	0.767	-0.026	[-0.071,	0.253	0.028	[-0.015,	0.203	-0.026	[-0.07,	0.259	-0.030	[-0.074,	0.177	-0.025	[-0.069,	0.276

							0.05]			0.019]			0.071]			0.019]			0.014]			0.02]	
			WC	0	0.0%	N/A	N/A	N/A	N/A	N/A	N/A	N/A	N/A	N/A	N/A	N/A	N/A	N/A	N/A	N/A	N/A	N/A	N/A
			Fasting glucose	†	†	-0.156	[-0.347, 0.035]	0.110	-0.053	[-0.195, 0.088]	0.459	0.001	[-0.158, 0.161]	0.988	-0.086	[-0.27, 0.097]	0.358	0.065	[-0.177, 0.308]	0.597	-0.068	[-0.27, 0.133]	0.506
			Hemoglobin A1C	1,097	0.5%	0.000	[-0.041, 0.04]	0.993	-0.044*	[-0.086, -0.002]	0.041	-0.003	[-0.043, 0.038]	0.891	-0.044*	[-0.086, -0.002]	0.040	-0.010	[-0.051, 0.031]	0.619	-0.043*	[-0.085, -0.001]	0.043
			Insulin	†	†	0.250	[-9.636, 10.135]	0.961	-0.066	[-3.727, 3.596]	0.972	0.086	N/A	N/A	0.071	N/A	N/A	-0.115	[-4.712, 4.482]	0.961	0.049	[-0.851, 0.95]	0.914
			BMI	170	0.1%	-0.036	[-0.222, 0.15]	0.706	0.087	[-0.093, 0.267]	0.341	-0.129	[-0.319, 0.061]	0.182	0.080	[-0.097, 0.257]	0.377	-0.119	[-0.291, 0.053]	0.176	0.105	[-0.075, 0.285]	0.254
			WC	0	0.0%	N/A	N/A	N/A	N/A	N/A	N/A	N/A	N/A	N/A	N/A	N/A	N/A	N/A	N/A	N/A	N/A	N/A	N/A
			Fasting glucose	†	†	-0.932*	[-1.827, -0.036]	0.041	1.431	[-0.158, 3.021]	0.078	-0.546	[-2.24, 1.147]	0.527	2.243	[-1.01, 5.495]	0.177	-0.794	[-2.028, 0.44]	0.207	2.353**	[0.717, 3.989]	0.005
			Hemoglobin A1C	†	†	-0.009	[-0.119, 0.101]	0.867	-0.019	[-0.132, 0.093]	0.737	-0.040	[-0.147, 0.066]	0.457	-0.028	[-0.141, 0.085]	0.631	0.020	[-0.077, 0.117]	0.692	-0.023	[-0.136, 0.089]	0.683
AFR	215	0.1%	Insulin	†	†	N/A	N/A	N/A	N/A	N/A	N/A	N/A	N/A	N/A	N/A	N/A	N/A	N/A	N/A	N/A	N/A	N/A	N/A
			BMI	165	0.1%	-0.034	[-0.218, 0.15]	0.719	-0.097	[-0.267, 0.074]	0.266	0.001	[-0.183, 0.185]	0.991	-0.097	[-0.267, 0.074]	0.267	-0.071	[-0.246, 0.104]	0.427	-0.088	[-0.259, 0.084]	0.318
AMR	185	0.1%	WC	0	0.0%	N/A	N/A	N/A	N/A	N/A	N/A	N/A	N/A	N/A	N/A	N/A	N/A	N/A	N/A	N/A	N/A	N/A	N/A

			Fasting glucose	†	†	N/A	N/A	N/A	N/A	N/A	N/A	N/A	N/A	N/A	N/A	N/A	N/A	N/A	N/A	N/A	N/A	N/A	N/A
			Hemoglobin A1C	†	†	-0.022	[-0.199, 0.155]	0.808	0.065	[-0.107, 0.236]	0.459	0.006	[-0.141, 0.153]	0.937	0.070	[-0.096, 0.237]	0.408	0.081	[-0.101, 0.264]	0.381	0.061	[-0.107, 0.229]	0.477
			Insulin	†	†	N/A	N/A	N/A	N/A	N/A	N/A	N/A	N/A	N/A	N/A	N/A	N/A	N/A	N/A	N/A	N/A	N/A	N/A
			BMI	69	<0.1 %	0.253	[-0.097, 0.603]	0.156	0.134	[-0.298, 0.566]	0.543	0.196	[-0.157, 0.549]	0.277	0.158	[-0.277, 0.594]	0.476	-0.102	[-0.436, 0.233]	0.551	0.164	[-0.276, 0.605]	0.465
			WC	0	0.0%	N/A	N/A	N/A	N/A	N/A	N/A	N/A	N/A	N/A	N/A	N/A	N/A	N/A	N/A	N/A	N/A	N/A	N/A
			Fasting glucose	†	†	N/A	N/A	N/A	N/A	N/A	N/A	N/A	N/A	N/A	N/A	N/A	N/A	N/A	N/A	N/A	N/A	N/A	N/A
			Hemoglobin A1C	†	†	0.093	[-0.154, 0.34]	0.462	-0.088	[-0.457, 0.28]	0.639	0.048	[-0.216, 0.312]	0.721	-0.110	[-0.479, 0.259]	0.559	0.604**	[0.178, 1.031]	0.006	-0.542**	[-0.95, -0.134]	0.009
EAS	75	<0.1 %	Insulin	0	0.0%	N/A	N/A	N/A	N/A	N/A	N/A	N/A	N/A	N/A	N/A	N/A	N/A	N/A	N/A	N/A	N/A	N/A	N/A
			BMI	†	†	0.038	[-0.295, 0.372]	0.822	-0.262	[-0.646, 0.121]	0.180	0.226	[-0.11, 0.562]	0.187	-0.240	[-0.59, 0.11]	0.179	-0.216	[-0.516, 0.084]	0.158	-0.271	[-0.637, 0.096]	0.148
			WC	0	0.0%	N/A	N/A	N/A	N/A	N/A	N/A	N/A	N/A	N/A	N/A	N/A	N/A	N/A	N/A	N/A	N/A	N/A	N/A
			Fasting glucose	0	0.0%	N/A	N/A	N/A	N/A	N/A	N/A	N/A	N/A	N/A	N/A	N/A	N/A	N/A	N/A	N/A	N/A	N/A	N/A
			Hemoglobin A1C	†	†	0.319***	[0.255, 0.383]	0.000	-0.051	[-0.395, 0.292]	0.770	-0.499***	[-0.761, -0.237]	0.000	0.143	[-0.096, 0.381]	0.241	0.265	N/A	N/A	-0.040	[-0.376, 0.297]	0.817
SAS	†	†	Insulin	0	0.0%	N/A	N/A	N/A	N/A	N/A	N/A	N/A	N/A	N/A	N/A	N/A	N/A	N/A	N/A	N/A	N/A	N/A	N/A
MID	†	†	BMI	†	†	0.531	[-0.61,	0.362	1.092	[-0.096,	0.072	2.374	[-3.207,	0.404	3.103	[-2.452,	0.274	-0.556	[-1.975,	0.443	1.234	[-0.369,	0.131

							1.672]			2.279]			7.955]			8.657]			0.863]			2.837]	
			WC	0	0.0%	N/A	N/A	N/A	N/A	N/A	N/A	N/A	N/A	N/A	N/A	N/A	N/A	N/A	N/A	N/A	N/A	N/A	N/A
			Fasting glucose	0	0.0%	N/A	N/A	N/A	N/A	N/A	N/A	N/A	N/A	N/A	N/A	N/A	N/A	N/A	N/A	N/A	N/A	N/A	N/A
			Hemoglobin A1C	†	†	N/A	s	N/A	N/A	N/A	N/A	N/A	N/A	N/A	N/A	N/A	N/A	N/A	N/A	N/A	N/A	N/A	N/A
			Insulin	0	0.0%	N/A	N/A	N/A	N/A	N/A	N/A	N/A	N/A	N/A	N/A	N/A	N/A	N/A	N/A	N/A	N/A	N/A	N/A

Fixed effects coefficients (β) from longitudinal analyses of PRS for sleep traits on BMI, WC, fasting glucose, hemoglobin A1C, insulin. PRS and all measurements were standardized to a mean of 0 and a standard deviation of 1. The linear mixed models were adjusted for Fitbit-derived sleep duration, ancestry, ethnicity, sex, smoking, obesity, and diabetes. Percentages are of the total 212,529 participants. Confidence intervals and p-values were unable to be computed for some parameters due to insufficient sample size or data variability. Significance levels: *p < 0.05, **p < 0.01, ***p < 0.001. † Counts suppressed per the All of Us Data and Statistics Dissemination Policy. ‡ Association test between Fitbit-derived sleep duration and health outcomes, with PRS included as a covariate..

eTable 7. PRS vs. sleep duration contribution analysis. We quantified the relative contributions of sleep duration (SD) compared to PRS-SD and PRS-SS using coefficients (β) from a linear mixed model for associations that were significant without sleep duration as a covariate but became non-significant after its inclusion, as shown in Tables 2 and 3.

	PRS-SS			PRS-SD		
	β_{PRS}	β_{SD}	$Contribution_{SD}$	β_{PRS}	β_{SD}	$Contribution_{SD}$
**Cross-sectional analyses**						
BMI	0.012	-0.089	98.2%	-0.018	-0.089	96.1%
WC	0.005	-0.056	99.2%	-0.001	-0.056	99.9%
Insulin	N/A	N/A	N/A	0.137	0.334	85.6%
**Longitudinal analyses**						
BMI	0.024	-0.021	44.0%	-0.041	-0.020	18.7%
Insulin	0.140	-0.039	7.1%	N/A	N/A	N/A

- Example formula used for linear mixed model: wc ~ PRS_SS + person_id + smoking + obesity + diabetes + sleep_duration + age_at_measurement + ancestry_pred_afr + ancestry_pred_amr + ancestry_pred_eas + ancestry_pred_mid + ancestry_pred_sas + ethnicity_Hispanic_or_Latino + sex_at_birth_Male
- $\text{Contribution}_{\text{sleep duration}} = \frac{{\beta_{SD}}^2}{{\beta_{PRS}}^2+{\beta_{SD}}^2} \times 100\ [\%]$
